# 2020 rankings for US PharmD programs, research, and overall quality

**DOI:** 10.1016/j.rcsop.2022.100169

**Published:** 2022-08-13

**Authors:** Lisa Lebovitz, Kimberly K. Daugherty, Margarita V. DiVall, Eric G. Boyce, Michael Rudolph

**Affiliations:** a University of Maryland School of Pharmacy, 20 North Pine Street, PH S303, Baltimore, MD 21201, United States of America; b Sullivan University College of Pharmacy and Health Sciences, 2100 Gardiner Lane, Louisville, KY 40205, United States of America; c Northeastern University School of Pharmacy, 120 Behrakis Life Sciences, Boston, MA 02115, United States of America; d Thomas J. Long School of Pharmacy, University of the Pacific, 3601 Pacific Avenue, Stockton, CA 95211, United States of America; e Lincoln Memorial University School of Medical Sciences, 6965 Cumberland Gap Parkway, Harrogate, TN 37752, United States of America

**Keywords:** Rankings, Program, Quality

## Abstract

**Background:**

US News and World Report (USNWR) publishes well-known rankings of graduate health programs. Medicine and nursing are ranked with weighted metrics using multiple criteria, and medical schools are ranked separately according to their focus (research or primary care). USNWR pharmacy school rankings are based on a single-question peer perception survey.

**Objective:**

The objective of this study was to develop a simple, transparent framework to rank US colleges and schools of pharmacy in overall quality and separately based on program quality and research quality, using data that are readily available to the academy.

**Methods:**

Data for three education quality and four research quality metrics were obtained for 2020. Each metric was standardized and ranked, and then each set was summed to determine separate ranks for education and research. Education and research scores were combined using equal weights to provide a single rank for overall quality. A sensitivity analysis was performed to determine the effect of assigning higher proportionate value to education, similar to USNWR medical school rankings.

**Results:**

Distinct ranks were produced for education, research, overall (education: research) 50:50, and overall 60:40. Sensitivity analysis suggests the more disproportionately the education and research factors are weighted, the more ranks change. Mid-ranked schools were most impacted when weightings changed due to relative strength in one factor and relative weakness in the other. When weighted 60:40, nine (7%) mid-ranked programs improved in rank, while 11 (11%) worsened in rank compared to the 50:50 model.

**Conclusion:**

Separately ranking education and research can highlight the diverse strengths of pharmacy schools. The proposed model is based on easily obtainable data and is easily reproducible, allowing for annual rankings. These rankings may be used by PharmD and PhD applicants when selecting schools and by pharmacy schools to benchmark true and aspirational peers.

## 1 Introduction

Nearly 25,000 individuals signed a change.org petition^1^ circulated in 2018 to protect the pharmacist profession, including tightening accreditation requirements such as a minimum 80% pass rate on the North American Pharmacist Licensure Examination (NAPLEX) for schools to maintain accreditation. The petitioners cited indicators of pharmacy school quality as accreditation status, US News and World Report (USNWR) rankings, and NAPLEX pass rates, all of which have their own limitations.^2^ USNWR rankings can be frustrating for colleges and schools of pharmacy and the general public because peer perception is the only criterion; they are based on an invalid rating scale with no criteria provided for the rating.^2^^,^^3^ The USNWR rankings also do not align with other studies of quality. Nau and colleagues^2^ found that when NAPLEX pass rates and USNWR rankings were compared side by side, many of the schools ranked in the top 10 in USNWR were not in the top 50 for NAPLEX, and schools with very high NAPLEX pass rates were not as highly ranked. Flawed rankings of academic programs and institutions may also result in members of those programs and institutions questioning their core identities and then reacting by developing inappropriate strategies and tactics in an effort to improve their rankings.^4^, ^5^, ^6^, ^7^

Many published rankings for universities and academic programs utilize inconsistent methodologies and quality criteria, and may not be based on objective data or any data, as is the case with USNWR pharmacy program rankings.^8^^,^^9^ However, USNWR medicine and nursing program rankings include objective metrics such as program selectivity and faculty resources (Table 1).^10^^,^^11^

**Table 1:** Criteria and weights for US News & World Report Best Medical Schools and Best Nursing Schools Rankings Formulas.^10^^,^^11^

Criteria and weighting of score	Med school research	Med school primary care	Nursing masters	Nursing DNP
Student selectivity	0.20	0.15	0.11	0.19
Faculty resources	0.10	0.15	0.24	0.26
Peer assessment score	0.15	0.25	0.40	0.40
Assessment score by residency directors	0.15	0.15		
Research activity	0.40	0	0.25	0.15
Primary Care Rate	0	0.30		

A study by Ried and Ried^12^ found that USNWR program rankings were higher if programs were older, were affiliated with an academic health center, were classified as research-intensive, or were members of a Power 5 athletic conference. The number of full-time faculty equivalents, pharmacy practice h-index, and research funding were also predictors of a program's USNWR ranking. Lastly, student PCAT comprehensive percentile and first-time NAPLEX pass rates were also found to influence rankings. Another study by Ried and Ried^13^ found that faculty and student attributes significantly impacted pharmacy school rankings. Faculty metrics included full-time faculty equivalents and research productivity, which were stronger predictors than student academic preparation or NAPLEX scores. The models in their study demonstrate the possibility of creating rankings using more objective data. However, compiling data from individual schools for this purpose is laborious.

Multiple available data sources reflect quality and could be used to determine pharmacy school rankings, including the American Association of Colleges of Pharmacy (AACP) Office of Institutional Research,^14^ the National Association of Boards of Pharmacy (NABP),^15^ and the American Society of Health-System Pharmacists (ASHP).^16^ AACP gathers information annually about pharmacy programs and students, full-time faculty, and external funding and makes it available to its members upon request. NABP publishes annual pass rates for the North American Pharmacist Licensure Examination (NAPLEX), and ASHP disseminates data annually to pharmacy school deans on PharmD graduates' placement in ASHP-accredited Postgraduate Year 1 (PGY1) residency programs.

The objective of this study was to create a simple model for ranking pharmacy schools that improves upon the USNWR pharmacy school rankings by utilizing metrics and data available without additional surveys, calculating ranks with a transparent and easily reproducible method, and considering educational and research strengths separately to reflect the breadth and variety of strengths among all pharmacy schools in the academy.

## 2 Methods

Seven indicators attributed to pharmacy school quality were identified from readily available sources. Three indicators represented PharmD program educational quality: student-to-faculty ratio (number of total PharmD students enrolled divided by the number of full-time faculty), NAPLEX pass rate for first-time candidates, and percentage of graduates matched to an ASHP-accredited residency program (number of PGY1 residency matches (both phases) divided by the number of PharmD graduates). The total PharmD students enrolled and the number of PharmD graduates were obtained from the AACP Profile of Pharmacy Students and Degrees Conferred tables.^17^^,^^18^ The number of full-time faculty was taken from the AACP Full-time Pharmacy Faculty Interactive Dashboard,^19^ the NAPLEX pass rates from NABP,^20^ and residency matches from ASHP email sent to pharmacy school deans. The other four indicators pertained to research: total research funding dollars, average award amount (total funding dollars divided by the number of funded faculty), the total number of principal investigators on NIH grants, and the number of PhDs conferred. The first three research variables were obtained from the AACP Funded Research Grant Institutional Rankings,^21^ while the fourth was drawn from the AACP Profile of Pharmacy Students, Degrees Conferred.^18^ While the USNWR medical school and nursing school rankings helped to inform our selection of education and research quality indicators, a number of indicators used by USNWR are not readily available for pharmacy schools, such as standardized admission test scores (PCAT instead of MCAT); undergraduate GPA; admissions selectivity (number of applicants offered admission); clinical practice participation; graduate outcomes; and other measures of faculty achievement.^10^^,^^11^

The dataset was cleaned using Microsoft Excel (Version 16.0.11126.20192; Microsoft, 2019) and IBM SPSS (Version 27; IBM, 2020). All variables were converted to a standard score (Z score) to place them on a common scale for calculating the rankings. One variable, student-to-faculty ratio, was reverse-coded so that the direction of the scale was consistent with the other variables (i.e., larger values would be associated with higher quality). When calculating the education rankings, schools missing one or more education variables were deleted listwise. Those schools that reported research funding to AACP but had no NIH investigators or PhDs conferred, or those schools with no research funding or program, were included in the research analysis but assigned zero values for those variables as appropriate.

A sensitivity analysis was performed to compare the effects of applying different weights to the education score and the research score when calculating the overall school ranks, similar to USNWR, that weights different metrics of the medicine and nursing rankings depending on school or program focus. The rank for each school was re-calculated using different 10-point increments for the weight of the education factor. For example, each school's total rank was calculated using a weight of 0 for the education factor and 100 for the research factor, followed by a weight of 10 for the education factor and 90 for the research factor, and so forth. A difference in rank (absolute value) was calculated for each school's rank at each increment compared to the rank produced from the 50:50 weighting scheme. Schools were divided into three groups containing an approximately equal number of schools, based on their overall rank using the 50:50 weighting scheme (i.e., group 1 - highest ranked third, group 2 - middle ranked third, group 3- lowest ranked third). The mean difference in rank was then compared for each group of schools and, overall, for the rank produced using each weighting scheme relative to the 50:50 approach. Results from the sensitivity analysis were also used to identify a second weighting scheme that could be useful and appropriate.

## 3 Results

Of the 141 US colleges and schools of pharmacy, four schools were excluded for incomplete or missing variables in both education and research, two schools were excluded as their accreditation had been withdrawn, and five schools had incomplete education and research data. There were 130 schools with complete education data for that ranking and 112 with complete data for the research ranking. By assigning zero values for missing research variables, education, research, and overall ranks for 130 schools were calculated in the final dataset.

**Fig. 1:**
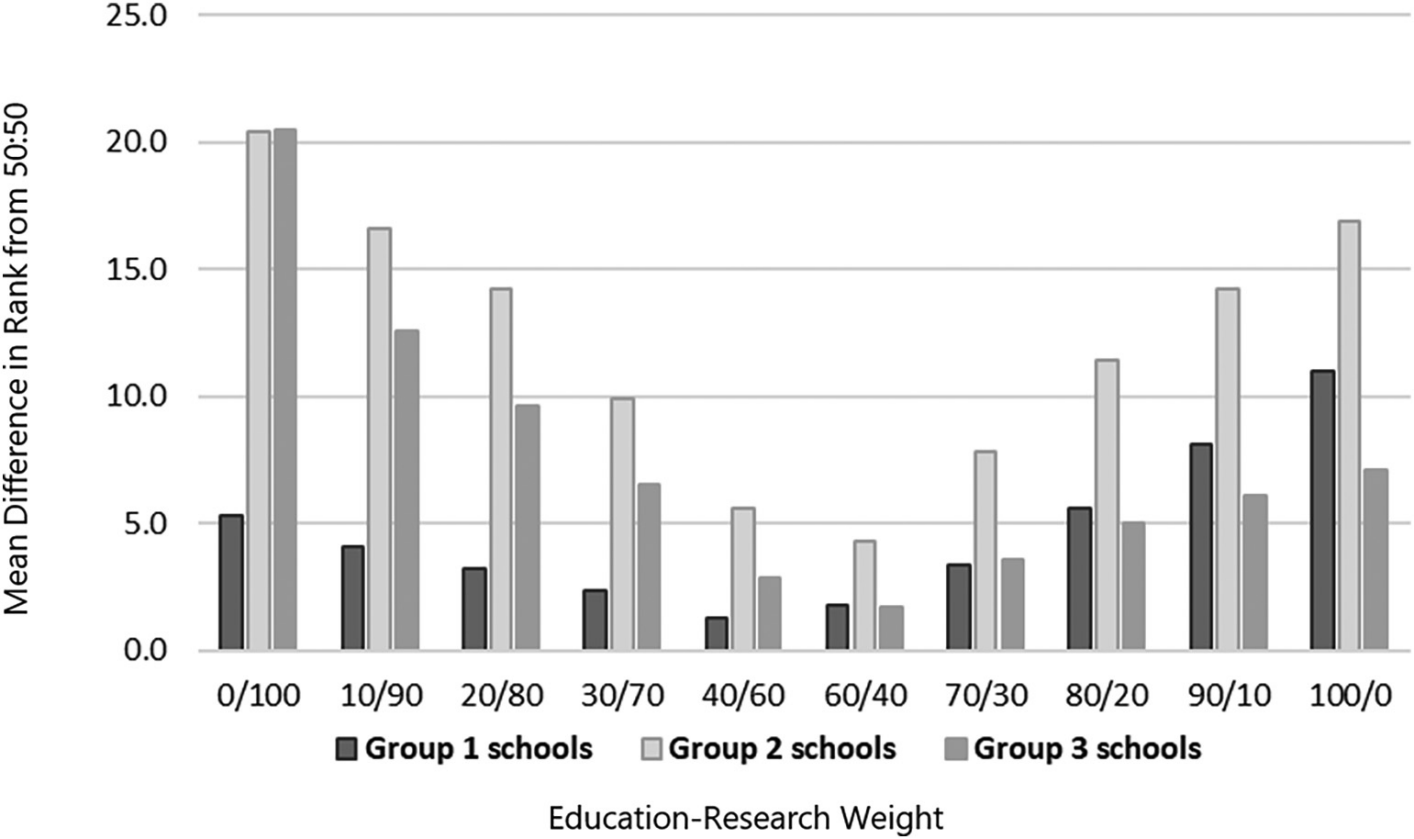
Mean differences in overall rank for different education:research weighting schemes, compared to 50/50 education:research weights. Note: Schools were divided into three groups containing an approximately equal number of schools, based on their overall rank using the 50:50 weighting scheme (i.e., group 1 - highest ranked third, group 2 - middle ranked third, group 3- lowest ranked third).

The sensitivity analysis (Fig. 1) suggests that the further from the 50:50 approach to weighting the education and research factors one deviates, the more dramatic the difference in the school rankings. For example, the mean difference in overall rank produced from the 50:50 scheme compared to the 60:40 is only 3.3 positions, whereas the difference from the 50:50 scheme compared to the 90:10 scheme is 11.1 positions. Additionally, the effects of changes in the weights applied to the education and research factors are not equal across the three groups of institutions. The greatest reshuffling of institutions appears to occur within the second (middle) group. An average change of 14.2 positions was observed for these institutions when education received a weight of 20 compared with changes of 3.2 and 9.6 for groups one and three. Institutions in the second group also changed an average of 11.4 positions when education received a weight of 80, compared with 5.6 and 5.0 for groups one and three. Calculated rankings for each pharmacy school included in this study compared to their USNWR ranking are presented in Table 2 using the 60:40 and 50:50 calculations.

**Table 2:** Overall, education, and research rankings for pharmacy schools 2020.

2020 Rankings	Overall 60:40	Overall 50:50	Edu-cation	*Re*-search	USNWR
University of California, San Francisco School of Pharmacy	1	1	1	1	2
University of North Carolina Eshelman School of Pharmacy	2	2	2	2	1
University of Michigan College of Pharmacy	3	3	3	7	3
University of Illinois at Chicago College of Pharmacy	4	4	10	4	7
University of Minnesota College of Pharmacy	5	5	5	12	3
The University of Utah College of Pharmacy	6	8	4	25	14
The University of Mississippi School of Pharmacy	7	7	7	13	24
Purdue University College of Pharmacy	8	6	20	5	7
The University of Texas at Austin College of Pharmacy	9	9	16	9	7
University of Nebraska Medical Center College of Pharmacy	10	13	9	19	28
University of Kentucky College of Pharmacy	11	11	18	10	6
University of Wisconsin-Madison School of Pharmacy	12	15	11	15	7
University of Pittsburgh School of Pharmacy	13	16	12	16	13
University of California, San Diego Skaggs School of Pharmacy & Pharmaceutical Sciences	14	17	6	36	18
University of Washington School of Pharmacy	15	12	27	8	7
The Ohio State University College of Pharmacy	16	14	24	11	7
University of Florida College of Pharmacy	17	10	47	3	5
The University of New Mexico College of Pharmacy	18	20	23	24	43
University of Houston College of Pharmacy	19	21	17	28	31
University of Colorado Anschutz Medical Campus Skaggs School of Pharmacy and Pharmaceutical Sciences	20	19	26	20	20
Virginia Commonwealth University at the Medical College of Virginia Campus School of Pharmacy	21	24	14	32	20
The University of Arizona College of Pharmacy	22	18	36	14	20
Medical University of South Carolina	23	31	8	51	31
University of Connecticut School of Pharmacy	24	27	19	31	29
Oregon State University College of Pharmacy	25	26	22	30	31
University of Maryland School of Pharmacy	26	22	32	22	14
University of Rhode Island College of Pharmacy	27	25	28	27	40
The University of Tennessee Health Science Center College of Pharmacy	28	28	38	21	20
Northeastern University Bouvé College of Health Sciences School of Pharmacy	29	30	35	26	31
The University of Kansas School of Pharmacy	30	29	39	23	24
University of Southern California School of Pharmacy	31	32	53	17	14
University of Montana College of Health Professions and Biomedical Sciences Skaggs School of Pharmacy	32	23	82	6	57
The University of Georgia College of Pharmacy	33	34	29	38	24
University at Buffalo The State University of New York School of Pharmacy & Pharmaceutical Sciences	34	33	42	29	14
South Dakota State University College of Pharmacy and Allied Health Professions	35	37	21	53	59
West Virginia University School of Pharmacy	36	35	31	39	31
Texas Tech University Health Sciences Center School of Pharmacy	37	36	40	34	46
The University of Iowa College of Pharmacy	38	38	33	46	18
Cedarville University School of Pharmacy	39	41	13	111	108
Thomas Jefferson University Jefferson College of Pharmacy	40	42	15	113	53
University of Arkansas for Medical Sciences College of Pharmacy	41	39	45	37	31
University of Cincinnati James L. Winkle College of Pharmacy	42	43	34	63	30
North Dakota State University College of Health Professions School of Pharmacy	43	40	57	35	59
Campbell University College of Pharmacy and Health Sciences	44	47	25	113	65
The University of Oklahoma College of Pharmacy	45	45	37	68	31
Wayne State University Eugene Applebaum College of Pharmacy and Health Sciences	46	44	54	43	43
University of Puerto Rico Medical Sciences Campus School of Pharmacy	47	46	43	57	71
Medical College of Wisconsin School of Pharmacy	48	52	30	113	
Northeast Ohio Medical University College of Pharmacy	49	50	41	72	71
University of Missouri-Kansas City School of Pharmacy	50	48	46	61	31
University of Maryland Eastern Shore School of Pharmacy and Health Professions	51	51	44	71	90
Auburn University Harrison School of Pharmacy	52	53	73	45	31
Washington State University College of Pharmacy	53	49	88	33	40
University of the Pacific Thomas J. Long School of Pharmacy & Health Sciences	54	55	65	56	59
St. John Fisher College Wegmans School of Pharmacy	55	62	49	103	90
Harding University College of Pharmacy	56	63	48	113	126
University of South Florida College of Pharmacy	57	57	61	70	68
Presbyterian College School of Pharmacy	58	65	50	113	119
Touro University - California College of Pharmacy	59	59	55	82	99
University of South Carolina College of Pharmacy	60	54	78	44	40
Samford University McWhorter School of Pharmacy	61	66	52	98	65
Pacific University School of Pharmacy	62	67	51	113	79
Ferris State University College of Pharmacy	63	61	60	77	68
The University of Toledo College of Pharmacy and Pharmaceutical Sciences	64	58	70	59	57
Idaho State University College of Pharmacy	65	64	63	76	59
St. John's University College of Pharmacy and Health Sciences	66	56	87	40	65
Southern Illinois University Edwardsville School of Pharmacy	67	69	56	102	68
Drake University College of Pharmacy and Health Sciences	68	70	58	99	46
The University of Findlay College of Pharmacy	69	75	59	107	108
Temple University School of Pharmacy	70	60	83	48	53
Western New England University College of Pharmacy	71	76	62	113	99
Concordia University Wisconsin School of Pharmacy	72	74	64	89	108
Butler University College of Pharmacy and Health Sciences	73	71	66	86	46
Western University of Health Sciences College of Pharmacy	74	73	68	84	71
University of the Incarnate Word Feik School of Pharmacy	75	77	67	90	108
Chapman University School of Pharmacy	76	68	90	49	99
Creighton University School of Pharmacy and Health Professions	77	79	71	87	46
Ohio Northern University College of Pharmacy	78	84	69	113	59
The University of Louisiana at Monroe College of Health and Pharmaceutical Sciences School of Pharmacy	79	78	81	66	79
Shenandoah University Bernard J. Dunn School of Pharmacy	80	80	79	74	79
East Tennessee State University Bill Gatton College of Pharmacy	81	85	72	104	71
Lipscomb University College of Pharmacy and Health Sciences	82	86	76	95	99
Wilkes University Nesbitt School of Pharmacy	83	87	74	113	79
Notre Dame of Maryland University School of Pharmacy	84	91	75	113	79
Florida Agricultural & Mechanical University College of Pharmacy and Pharmaceutical Sciences	85	72	101	42	79
Rosalind Franklin University of Medicine and Science College of Pharmacy	86	89	77	91	99
University of the Sciences Philadelphia College of Pharmacy	87	83	91	65	46
Mercer University College of Pharmacy	88	81	95	58	53
Duquesne University School of Pharmacy	89	82	100	50	43
High Point University Fred Wilson School of Pharmacy	90	92	86	83	
Belmont University College of Pharmacy	91	95	80	113	90
Southwestern Oklahoma State University College of Pharmacy	92	94	89	85	90
Marshall University School of Pharmacy	93	93	93	81	79
Texas A & M University Health Science Center Irma Lerma Rangel College of Pharmacy	94	88	103	52	46
Sullivan University College of Pharmacy	95	98	84	113	119
Palm Beach Atlantic University Lloyd L. Gregory School of Pharmacy	96	99	85	113	119
University of North Texas System College of Pharmacy	97	90	105	47	90
Loma Linda University School of Pharmacy	98	101	92	109	90
Keck Graduate Institute (KGI) School of Pharmacy	99	97	98	67	108
Touro New York College of Pharmacy	100	102	94	100	128
Howard University College of Pharmacy	101	96	104	54	75
Albany College of Pharmacy and Health Sciences School of Pharmacy and Pharmaceutical Sciences	102	100	102	73	59
St. Louis College of Pharmacy	103	103	96	113	46
Manchester University College of Pharmacy, Natural and Health Sciences	104	105	97	112	108
Midwestern University Chicago College of Pharmacy	105	107	99	113	75
Nova Southeastern University College of Pharmacy	106	106	109	64	79
Texas Southern University College of Pharmacy and Health Sciences	107	104	113	55	108
Long Island University Arnold and Marie Schwartz College of Pharmacy and Health Sciences	108	108	116	60	79
Roseman University of Health Sciences College of Pharmacy	109	110	106	113	108
Midwestern University College of Pharmacy-Glendale	110	111	107	113	75
Regis University School of Pharmacy	111	113	108	113	90
University of Hawaii at Hilo Daniel K. Inouye College of Pharmacy	112	114	110	94	79
Fairleigh Dickinson University School of Pharmacy	113	115	112	97	119
MCPHS University School of Pharmacy - Boston	114	112	118	62	75
Wingate University School of Pharmacy	115	116	111	110	90
Xavier University of Louisiana College of Pharmacy	116	109	122	41	90
California Northstate University College of Pharmacy	117	117	114	101	128
Roosevelt University College of Pharmacy	118	118	115	104	119
Appalachian College of Pharmacy	119	119	117	113	119
Marshall B. Ketchum University College of Pharmacy	120	120	119	104	
Lake Erie College of Osteopathic Medicine (LECOM) School of Pharmacy	121	121	120	113	99
University of Saint Joseph School of Pharmacy	122	122	121	113	117
University of Charleston School of Pharmacy	123	123	124	79	117
West Coast University School of Pharmacy	124	124	123	113	132
South College School of Pharmacy (TN)	125	125	125	113	134
MCPHS University School of Pharmacy - Worcester	126	126	126	92	99
University of New England College of Pharmacy	127	127	127	113	99
Philadelphia College of Osteopathic Medicine School of Pharmacy	128	128	128	108	99
Larkin University College of Pharmacy	129	130	129	113	
Chicago State University College of Pharmacy	130	129	130	88	128

Note: Blanks indicate schools that had no 2020 USNWR rank.

When comparing the overall rankings and USNWR, the mean difference in ranking (absolute value) was 15.6 positions for the 50:50 model and 16.5 positions for the 60:40 model, respectively. The mean difference in ranking between the education ranking and USNWR was 20.6 positions compared to 17.0 between the research ranking and USNWR. Given the inclusion of 126 schools with education, research, and USNWR rankings, the average shifting of between 15.6 and 20.6 positions between the various rankings and USNWR is considerable. Consistent with early comparisons of applying different weighting schemes within the current rankings, smaller differences were observed between the 60:40 model ranking and USNWR ranking for the top group of schools (a mean change of 10.3 positions) compared with the middle and lower groups (21.1 and 18.0 positions).

## 4 Discussion

The USNWR ranks nursing graduate programs^10^ and medical schools^11^ more objectively than pharmacy by using several objective indicators of program quality, in addition to a peer assessment score. Separate rankings are calculated for research-focused medical schools using a weighted average of 12 indicators, including research activity, and for primary care schools using seven indicators, including the proportion of medical graduates entering primary care specialties. Both rankings include admissions selectivity and student-to-faculty ratios (Table 1). Doctor of Nursing Practice (DNP) and Master of Nursing (MS) rankings use a weighted average of 14 indicators; seven are used in both frameworks (four research activity and three faculty quality), and the other seven indicators are specific to each degree (Table 1).

Although USNWR uses quality metrics for medicine, nursing, and undergraduate rankings, pharmacy programs are ranked purely on peer perception.9., 10., 11. Every four years, a limited number of surveys are sent to each fully accredited pharmacy program in good standing. The USNWR pharmacy program survey asks respondents to consider all factors that relate to excellence in each program, such as curriculum, scholarship and research, and quality of faculty and graduates, to evaluate each program. Respondents rate each school with a single checkmark, 1 = marginal, 2 = adequate, 3 = good, 4 = strong, 5 = outstanding, and “don't know” if the respondent does not have enough knowledge to rate a program.^9^

Popular rankings may influence perceptions of potential student applicants, dean and faculty applicants, preceptors, patients, funding agencies, donors, collaborators and partners, and other entities. Therefore, it is important to align ranking systems with measures of program quality that address the interests of those using the results to make decisions. Studies found that USNWR pharmacy program rankings correlate strongly with total grant funding, NIH and non-NIH grant funding, years in existence, and association with an academic medical center.^22^^,^^23^ Faculty publication rates were also significantly correlated in one study. Therefore, perceptions in the USNWR pharmacy program rankings appear to favor the longer-established and research-intensive schools while potentially failing to recognize educational quality across the academy.

In this novel study, education and research were initially assigned equal weight in the overall ranking calculation. The authors then debated whether to assign slightly greater weight to education in the overall calculation; education is the primary goal of all schools, and the main audience of program ranking is the prospective applicants interested in educational quality. The study team explored this issue using sensitivity analysis of equal versus unequal weights between the two categories.

The sensitivity analysis revealed the impact of the weightings of the academic and research components on the overall rankings. The baseline analysis used education-research weightings of 50:50. As noted in the sensitivity analysis, an education-research weighting of 60:40 would have resulted in some changes in the rankings. However, further deviation from the 50:50 weighting resulted in a higher level of deviation in the ranks. This deviation was lowest in the schools initially categorized in group 1 (highest ranked at 50:50) and highest for those schools initially determined to fall in group 2. Schools in group 1 displayed more relative strengths in both major components (education and research); schools in group 2 displayed strength in one component but weakness in the other, and schools in group 3 displayed more relative weakness in both major components.

To that end, the education-research weighting of 50:50 or 60:40 in determining the overall ranks is recommended. Those weightings maintain the importance of research for the academy. Additionally, we observe that the education-research weighting of 60:40 is consistent with the USNWR process for ranking research-intensive medical schools and that USNWR calculates rankings for primary care medical schools, nursing masters, and nursing DNP programs with even greater proportionate weight placed on the education variables (Table 1).^10^^,^^11^

This research aimed to develop a simple data-driven ranking of pharmacy schools similar to other healthcare professions using readily available metrics that reflect the quality of research and education. An empirical framework was developed using objective data obtained from AACP, ASHP, or the public domain; the metrics selected were similar to those used in USNWR rankings for medical and nursing programs (Table 1).^10^^,^^11^ Other educational quality measures identified by deans were not included, partly due to difficulty obtaining reliable data: public and patient care service, stakeholder feedback, testing, student success, and curriculum.^24^ This framework also excluded other factors correlated with the USNWR rankings, such as the number of years in existence and association with an academic medical center,^23^ which may underestimate academic program quality in newer pharmacy schools.

Using standardized objective measures of pharmacy school quality, such as those used in this paper, could inform the development and implementation of strategic plan goals and aid in selecting true and aspirant peers for benchmarking. Pharmacy schools could then target specific metrics for improvement and resource allocation to be more appealing to a potential student or faculty applicants. Further, as the data utilized for analysis are updated annually, rankings can be calculated annually, providing a real-time metric of success to pharmacy institutions.

The methods to develop these rankings provide a general framework that can be easily replicated or adapted for future data. Unlike the reputational scores used in USNWR rankings that may be slow to change, calculated rankings will reflect significant system-wide changes in current and future quality measures (i.e., board passage rates, NIH and other funding).

There are potentially numerous limitations to any ranking system. The researchers attempted to minimize those limitations by carefully selecting quality-based metrics with data available to pharmacy schools or in the public domain. However, not all possible metrics associated with the academic program or research quality were included. For example, several schools received a research score of zero in funding metrics, although there are other forms of scholarship, such as publications in peer-reviewed journals. Additional indicators would strengthen the results, such as measures for the provision of patient care or community service, the pursuit of fellowships or graduate education, tuition or debt burden, entry-level salaries, and types of employment, although gathering these data would be laborious endeavors as they are not readily available.

Using a single year of data to calculate rankings presents another limitation because results are sensitive to year-to-year fluctuations, although this is common practice with USNWR and others. This may be mitigated by including multiple indicators; however, a multi-year average may be preferable in future ranking calculations. Another concern is the age of the data used. Most organizations, such as AACP and ASHP, compile data for the preceding academic year, then clean, analyze, and publish those data. As such, even a simple ranking model is based on data close to two years old.

## 5 Conclusion

This framework suggests a relatively easy and more objective approach to pharmacy school rankings using distinct quality dimensions in education and research. A focus on both academic program quality and research-based quality may be useful to the academy due to its inclusivity. Future researchers may consider how much emphasis should be assigned to each dimension and are encouraged to identify additional data sources and quality metrics, including those that are proprietary or collected through surveys or open records requests. Pharmacy schools may benefit from using this study's metrics to develop strategic plans for improvement and benchmark with peer institutions. Given the discrepancies between this model-driven approach and the USNWR peer perception scoring system, deans and academy leaders should advocate for a new ranking system or changes to the existing USNWR *Best Pharmacy Schools*.

## Funding sources

This research did not receive any specific grant from funding agencies in the public, commercial, or not-for-profit sectors.

## Declaration of Competing Interest

None.
